# Evaluation of a cumulative prognostic score based on the systemic inflammatory response in patients undergoing potentially curative surgery for colorectal cancer

**DOI:** 10.1038/sj.bjc.6601757

**Published:** 2004-03-30

**Authors:** K Canna, D C McMillan, R F McKee, A-M McNicol, P G Horgan, C S McArdle

**Affiliations:** 1 University Department of Surgery, Glasgow Royal Infirmary, Alexandra Parade, Glasgow G31 2ER, UK; 2 University Department of Pathology, Glasgow Royal Infirmary, Glasgow G31 2ER, UK

**Keywords:** colorectal cancer, Dukes' stage, C-reactive protein, prognostic score, survival

## Abstract

The value of combining Dukes' stage and C-reactive protein to form a cumulative prognostic score was assessed in 147 patients undergoing potentially curative resection for colorectal cancer. The cancer-specific survival rates at 3 years for patients with a cumulative prognostic score of 0, 1 and 2 were 100, 77 and 40%, respectively (HR 4.76, 2.78–8.15, *P*<0.001).

Colorectal cancer is the second commonest cause of cancer death in North America and Western Europe. Each year in the UK, there are approximately 27 000 new cases and approximately 18 000 deaths attributable to the disease. Overall survival is poor; even in those who undergo potentially curative resection, only half survive 5 years (McArdle *et al*, 2003).

The ideal prognostic score for patients undergoing potentially curative resection of a primary colorectal cancer should clearly distinguish those who will eventually succumb to the disease from those who are cured. While Dukes' stage has been widely used, it fails to provide clear separation between these groups. Alternative factors that would provide additional information to that of Dukes' staging are therefore required.

There is increasing evidence that the presence of a systemic inflammatory response, as evidenced by elevated circulating concentrations of C-reactive protein, is associated with increased recurrence and poor survival in patients undergoing potentially curative surgery for colorectal cancer (McMillan *et al*, 1995,^2003^; Nozoe *et al*, 1998; Nielsen *et al*, 2000; Wigmore *et al*, 2001; Chung and Chang, 2003). However, some of these studies have questioned whether C-reactive protein has prognostic value independent of conventional pathological criteria including Dukes' stage (Wigmore *et al*, 2001; Chung and Chang, 2003).

The aim of the present study was to assess whether or not an elevated circulating C-reactive protein concentration has prognostic value independent of conventional clinicopathological criteria in patients undergoing potentially curative resection for colorectal cancer.

## PATIENTS AND METHODS

### Patients

Patients with Dukes' B and C colorectal cancer, who, on the basis of laparotomy findings and pre-operative computed tomography, underwent potentially curative resection between January 1997 and September 2001 in a single surgical unit at the Glasgow Royal Infirmary, were included in the study. Patients who had pre-operative radiotherapy were excluded from the study.

Prior to surgery, a blood sample was taken for routine laboratory measurement of albumin and C-reactive protein. At this time, no patient showed clinical evidence of tumour metastases, infection, or other inflammatory conditions. The tumours were staged using conventional Dukes' classification (Dukes and Bussey, 1958). All patients were followed-up at a single specialist colorectal cancer clinic.

The study was approved by the local ethical committee.

### Statistics

Data are presented as median and range. Comparisons between groups of patients were carried out using contingency table analysis (*χ*^2^). Grouping of the variables age, albumin and C-reactive protein was carried out using standard thresholds (Scottish Cancer Intelligence Unit, 2000; O'Gorman *et al*, 2000; Forrest *et al*, 2003).

Survival (cancer-specific) analysis was performed using the Cox proportional hazard model. Deaths up to September 2003 have been included in the analysis. Multivariate survival analysis was performed using a stepwise backward procedure to derive a final model of the variables that had a significant independent relationship with survival. To remove a variable from the model, the corresponding *P*-value had to be greater than 0.10. Analysis was performed using SPSS software (SPSS Inc., Chicago, IL, USA).

## RESULTS

The baseline clinicopathological characteristics of the patients (*n*=147) who underwent potentially curative surgery for colorectal cancer are shown in Table 1

**Table 1:** Clinicopathological characteristics in patients with colorectal cancer: univariate survival analysis

	**Patients (*n*=147)**	**Hazard ratio (95% CI)**	***P*-value**
Age group (<65/65–74/⩾75)	46/44/57	2.33 (1.42–3.83)	<0.001
Sex (male/female)	78/69	2.11 (1.03–4.36)	0.043
Site (colon/rectum)	105/42	1.38 (0.60–3.21)	0.451
Dukes stage (B/C)	91/56	5.07 (2.33–11.02)	<0.001
Albumin (<35/⩾35 g l^−1^)	31/116	0.86 (0.33–2.26)	0.766
C-reactive protein (<10/⩽10 mg l^−1^)	94/53	5.02 (2.35–10.74)	<0.001

*Tumour characteristics*			
Diameter (tertiles)	49/49/49	1.02 (0.67–1.56)	0.927
Ulceration (no/yes)	72/75	1.20 (0.59–2.43)	0.611
Differentiation (well/moderate/poor)	18/116/13	1.57 (0.70–3.50)	0.274
Lymphatic invasion (negative/positive)	124/22	1.83 (0.79–4.26)	0.158
Venous invasion (negative/positive)	118/28	2.43 (1.14–5.16)	0.021
			
Adjuvant therapy (no/yes)	116/31	1.13 (0.49–2.62)	0.779

. Approximately one-third of patients were aged 75 or over. The majority had colonic tumours, were Dukes' stage B and had moderately differentiated tumours. In all, 53 (36%) patients had an elevated C-reactive protein concentration prior to surgery. A total of 31 patients received 5-FU-based chemotherapy.

The minimum follow-up was 24 months; the median follow-up of the survivors was 56 months. During this period 45 patients died, 31 patients of their cancer and 14 of intercurrent disease.

On univariate analysis, increased age (*P*<0.001), sex (*P*<0.05), Dukes' stage (*P*<0.001), elevated circulating C-reactive protein concentrations (*P*<0.001) and venous invasion (*P*<0.05) were associated with poor cancer-specific survival. On multivariate analysis, including the above variables, age (HR 1.97, 95% CI 1.21–3.23, *P*=0.008), Dukes stage (HR 5.47, 95% CI 2.50–11.99, *P*<0.001) and C-reactive protein (HR 4.27, 95% CI 1.94–9.41, *P*<0.001) were significantly associated with cancer-specific survival.

Since the magnitude of the covariates of Dukes' stage (1.70) and C-reactive protein (1.45) were similar, this indicates that a one unit increase in C-reactive protein had approximately the same relative risk as a one unit increase in pathological stage and that they could be simply added to form a prognostic score. Such a cumulative prognostic score was therefore constructed by assigning one point for each of the following criteria: Dukes' stage C and C-reactive protein >10 mg l^−1^.

The relationship between stage, C-reactive protein concentration, the cumulative prognostic score and cancer-specific mortality is shown in Table 2

**Table 2:** Prognostic score following curative resection for colorectal cancer

**Dukes' stage**		**C-reactive protein**				
**Stage**	**Score**	**(mg l^−1^)**	**Score**	**Cumulative score**	**Patients (*n*)**	**3-year survival rate (%)**
B	0	⩽10	0	0	61	100
		>10	1	1	30	80
C	1	⩽10	0	1	33	77
		>10	1	2	23	40

Cumulative score obtained by adding scores for Dukes' stage and C-reactive protein.

**Figure 1:**
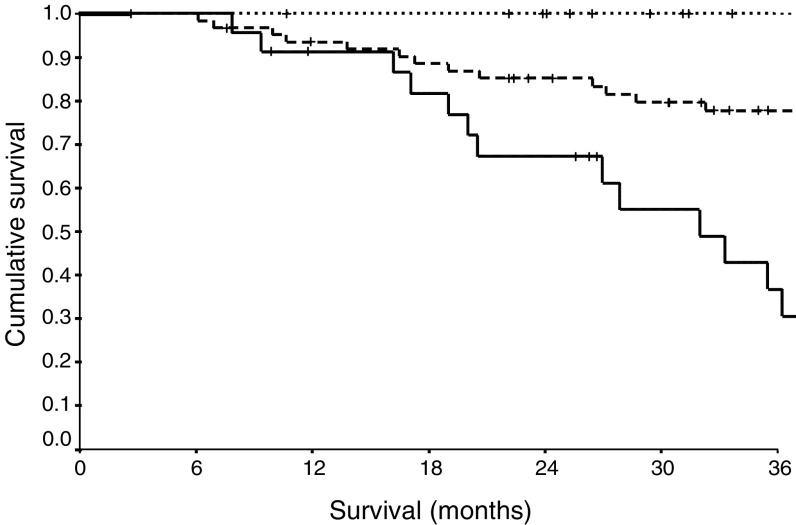
The relationship between the cumulative prognostic score (0 . . . ., 1- - , 2—) and cancer-specific survival following potentially curative surgery for colorectal cancer.

. The relationship between the cumulative prognostic score and cancer-specific survival is shown in Figure 1

. The cancer-specific survival rates at 3 years for patients with a cumulative prognostic score of 0, 1 and 2 were 100, 77 and 40%, respectively (HR 4.76, 95% CI 2.78–8.15, *P*<0.001).

## DISCUSSION

Several studies have shown that elevated circulating C-reactive protein concentrations are associated with poor survival in patients with colorectal cancer (McMillan *et al*, 1995; Nozoe *et al*, 1998; Nielsen *et al*, 2000; Wigmore *et al*, 2001; McMillan *et al*, 2003; Chung and Chang, 2003). However, the relationship between C-reactive protein concentrations and conventional clinicopathological criteria is not clear, since some of the above studies have included patients with Dukes A tumours who were unlikely to progress and patients with Dukes D tumours who had already progressed (Wigmore *et al*, 2001; Chung and Chang, 2003). This is likely to have confounded the assessment of the prognostic value of an elevated circulating C-reactive protein concentration.

In the present study, both Dukes' stage and C-reactive protein concentrations were independently associated with cancer-specific survival. These results are consistent with those of Nielsen and co-workers (2000) who, in a cohort of almost 400 patients undergoing resection for Dukes B and C tumours, also demonstrated that C-reactive protein was a Dukes' stage independent prognostic factor. The mechanism by which a systemic inflammatory response might influence cancer survival is not clear. However, it is known that as part of the systemic inflammatory response, there is a release of pro-inflammatory cytokines and growth factors which may promote tumour growth (Abramovitch *et al*, 1999) and compromise immune function (Coussens and Werb, 2002).

In the present study, when C-reactive protein concentrations were combined with Dukes' stage to form a new prognostic score, the combined score improved the prediction of cancer-specific survival. The addition of C-reactive protein differentiated between low- and high-risk Dukes' B and low- and high-risk Dukes' C patients. Cancer-specific survival at 3 years ranged from 100% in patients with Dukes' B tumours and a normal C-reactive protein concentration to 40% in patients with Dukes' C tumours and an elevated C-reactive protein concentration. Cancer-specific survival in patients with Dukes' B tumours and an elevated C-reactive protein concentration was similar to that of patients with Dukes' C tumours and a normal C-reactive protein concentration. Therefore, this cumulative prognostic score may be useful in identifying high-risk Dukes' B patients suitable for adjuvant therapy.

The results of the present study indicate that this simple prognostic score, which reflects both the contribution of the tumour and the host response, differentiates between low- and high-risk Dukes' B and C tumours in patients undergoing potentially curative resection for colorectal cancer.
